# Improved Flexural Properties of Experimental Resin Composites Functionalized with a Customized Low-Sodium Bioactive Glass

**DOI:** 10.3390/polym14204289

**Published:** 2022-10-12

**Authors:** Matej Par, Laura Plančak, Lucija Ratkovski, Tobias T. Tauböck, Danijela Marovic, Thomas Attin, Zrinka Tarle

**Affiliations:** 1 Department of Endodontics and Restorative Dentistry, School of Dental Medicine, University of Zagreb, Gunduliceva 5, 10000 Zagreb, Croatia; 2 School of Dental Medicine, University of Zagreb, Gunduliceva 5, 10000 Zagreb, Croatia; 3 Department of Conservative and Preventive Dentistry, Center of Dental Medicine, University of Zurich, Plattenstrasse 11, 8032 Zurich, Switzerland

**Keywords:** dental resin composite, experimental composite, bioactive glass, flexural strength, flexural modulus, reliability analysis, degree of conversion

## Abstract

This study evaluated the flexural properties of an experimental composite series functionalized with 5–40 wt% of a low-Na F-containing bioactive glass (F-series) and compared it to another experimental composite series containing the same amounts of the conventional bioactive glass 45S5 (C-series). Flexural strength and modulus were evaluated using a three-point bending test. Degree of conversion was measured using Fourier-transform infrared spectroscopy. Weibull analysis was performed to evaluate material reliability. The control material with 0 wt% of bioactive glass demonstrated flexural strength values of 105.1–126.8 MPa). In the C-series, flexural strength ranged between 17.1 and 121.5 MPa and was considerably more diminished by the increasing amounts of bioactive glass than flexural strength in the F-series (83.8–130.2 MPa). Analogously, flexural modulus in the C-series (0.56–6.66 GPa) was more reduced by the increase in bioactive glass amount than in the F-series (5.24–7.56 GPa). The ISO-recommended “minimum acceptable” flexural strength for restorative resin composites of 80 MPa was achieved for all materials in the F-series, while in the C-series, the materials with higher bioactive glass amounts (20 and 40 wt%) failed to meet the requirement of 80 MPa. The degree of conversion in the F-series was statistically similar or higher compared to that of the control composite with no bioactive glass, while the C-series showed a declining degree of conversion with increasing bioactive glass amounts. In summary, the negative effect of the addition of bioactive glass on mechanical properties was notably less pronounced for the customized bioactive glass than for the bioactive glass 45S5; additionally, mechanical properties of the composites functionalized with the customized bioactive glass were significantly less diminished by artificial aging. Hence, the customized bioactive glass investigated in the present study represents a promising candidate for functionalizing ion-releasing resin composites.

## 1. Introduction

Contemporary resin composites are characterized by favorable mechanical properties and excellent esthetics, which makes them the materials of choice for multiple indications in restorative dentistry, prosthodontics, pedodontics, and orthodontics [1]. An important shortcoming that has still not been overcome despite all advances is the high risk of developing a recurrent carious lesion at the tooth/restoration margin. This margin is never perfectly sealed due to polymerization contraction [2], and the risk of recurrent (secondary) caries limits the service life of these otherwise successful materials. Overcoming the issue of secondary caries would improve the long-term prognosis of resin composite restorations and broaden the indications for using resin composite materials, e.g., to the group of patients with suboptimal oral hygiene [3]. Another major group of restorative materials is glass ionomer cements, which undergo the acid–base reaction to form poly-salt hydrogels in which unreacted glass particles are embedded [4].

In efforts to decrease the susceptibility of resin composites to secondary caries by rendering them “remineralizing” or “bioactive”, various modifications have been attempted, from formulating hybrid resin composite/glass ionomer materials [5] or including the pre-reacted glass ionomer as part of the filler [6], to modifying the conventional glass-filled dimethacrylate resin by including ion-releasing fillers [7,8]. Extensive research of this approach has shown that various fillers (monocalcium, dicalcium, tricalcium, and octacalcium phosphate; amorphous calcium phosphate, hydroxyapatite, and bioactive glasses) can be used to release remineralizing ions that could shift the demineralization/remineralization equilibrium towards remineralization, thus reducing the incidence of secondary caries [1,9,10]. Unlike reinforcing fillers, the ion-releasing fillers are typically unsilanized to allow water access to the particle surface, which facilitates ion release. Incorporating the unsilanized and water-soluble particles in the resin composite usually compromises the mechanical properties of the composite material [11,12,13]. Instead of chemically bonding to the polymer network and distributing stresses like silanized glass particles, the ion-releasing fillers passively occupy the spaces within the polymer, without mechanically strengthening the material [11]. Upon water exposure, the particles gradually dissolve, further impairing mechanical properties. As in polymer composites used for various other applications [14,15,16,17,18,19], the mechanical properties of dental restorative composites are crucial for withstanding mechanical loads throughout the service life of the restoration.

In vitro mechanical testing of fundamental properties such as flexural strength and modulus enables studying various compositional adjustments in experimental materials under controlled conditions. While neither of the in vitro test methods fully simulates the multidirectional and repeated loading during mastication, flexural testing has been argued to be the most relevant among macromechanical tests as it simultaneously subjects the specimen to tensile, compressive, and shear forces [20]. Hence, flexural testing one day after material curing has been included in the basic array of tests recommended for restorative resin composites by the International Standards Organization (ISO). As the mechanical properties of resin composites are known to develop significantly with aging, introducing longer post-cure periods [21] and artificial aging [22] before mechanical testing better simulates the material behavior under oral conditions. Using artificial aging is especially important for ion-releasing resin composites, which are expectedly more affected by water-induced degradation than conventional resin composites [23].

Bioactive glasses (BGs) are being extensively investigated as functional fillers in restorative resin composites, owing to their potential to release ions [24], neutralize acid [25], and precipitate calcium phosphate [26]. Since the “bioactivity” and solubility of an individual BG composition can be adjusted by compositional modifications [27], a customized BG has been prepared based on modifying the conventional 45S5 composition by reducing its sodium content and introducing fluoride while keeping network connectivity unchanged [25]. The obtained BG was included as a functional filler in experimental resin composites, which showed ion release (calcium, phosphate, and fluoride) [28,29], acid-neutralization [25], and the potential to protect enamel and dentin against acid-induced demineralization [30,31]. In the aforementioned studies, the experimental composites with the low-Na F-containing BG consistently showed lower reactivity compared to the composites functionalized with BG 45S5, expectedly, due to the higher solubility of BG 45S5. It was hence hypothesized that the more stable BG would provide better mechanical properties than BG 45S5, which was previously demonstrated to significantly diminish the mechanical properties of experimental resin composites [13].

The present study aimed to evaluate the flexural properties of an experimental series of resin composites functionalized with 5–40 wt% of a low-Na F-containing BG. Another experimental composite series functionalized with the same amounts of BG 45S5 was used for comparison. The hypotheses were that: (I) composites with the low-Na F-containing BG would show better flexural properties than composites with the corresponding amount of BG 45S5, (II) increasing the BG amount would diminish flexural properties regardless of the BG type, and (III) artificial aging would affect flexural properties.

## 2. Materials and Methods

### 2.1. Experimental Composite Materials

The conventional BG 45S5 and a customized low-Na F-containing BG were prepared via melt-quench route and ground using the same standardized procedure in order to ensure comparable particle geometry for both BG formulations [25]. The customized low-Na F-containing BG was designed to have a theoretical network connectivity of 2.1, which was similar to that of BG 45S5. As reinforcing (inert) fillers, silanized barium glass and silica were used. A detailed composition of filler particles is shown in Table 1.

The resin system was a 60:40 wt% mixture of bisphenol-A-glycidyldimethacrylate (bis-GMA, Merck, Darmstadt, Germany) and triethylene glycol dimethacrylate (TEGDMA, Merck). The properties of methacrylate resins are shown in Table 2. The photoinitiator system consisted of camphorquinone (0.2 wt%; Merck) and ethyl-4-(dimethylamino) benzoate (0.8 wt%; Merck). The bis-GMA/TEGDMA comonomer blend and photoinitiator system were mixed in a dark bottle using a magnetic stirrer for 48 h. The obtained photoactivated resin system was mixed with inorganic fillers using a dual asymmetric centrifugal mixing system (Speed Mixer TM DAC 150 FVZ, Hauschild & Co. KG, Hamm, Germany) at 2000 rpm for 5 min, followed by deaeration in vacuum for 48 h.

Two series of experimental resin composites functionalized with 5–40 wt% of BG were prepared (Table 3). The C-series contained conventional BG 45S5, while the F-series contained the low-Na F-containing BG. The rest of the inorganic content was composed of reinforcing fillers, i.e., silanized barium glass and silica, up to a total of 70 wt%. The Control composite contained only 70 wt% of reinforcing fillers.

In addition to the experimental resin composites, a commercial bis-GMA/TEGDMA-based resin composite Charisma Classic (shade: A2; Kulzer, Hanau, Germany) was used as a reference.

### 2.2. Mechanical Testing

Flexural strength (FS) and flexural modulus (FM) were measured using a three-point bending test according to NIST 4877. This testing protocol features similar mechanics as flexural testing according to ISO 4049, with the different effective volumes of the material under load, due to the lower span between supports (12 mm for NIST or 20 mm for ISO). As both FS and FM have been demonstrated to depend on the inter-support span, the NIST-recommended value of 12 mm was chosen as more clinically realistic [32].

Rectangular specimens of 2 × 2 × 16 mm were prepared by casting the uncured composite in custom-made Teflon split-molds, covering mold openings with polyethylene terephthalate (PET) foil, and pressing the mold between two thick (5 mm) glass plates. After removing the glass plate, the specimens were light-cured using a wide spectrum LED curing unit Bluephase PowerCure (Ivoclar Vivadent, Schaan, Liechtenstein) with a radiant exitance of 1050 mW/cm^2^, as measured with a calibrated and NIST-referenced UV-Vis spectrophotometer system (MARC; BlueLight Analytics, Halifax, NS, Canada), using three successive light exposures, each lasting 20 s and not overlapping with the previous irradiated section for more than 1 mm. An additional three successive light exposures (20 s each) were used on the opposite side of the specimens in order to ensure a thorough light-cure, as recommended by the ISO 4049 protocol. Specimens were taken out of the molds and gently ground on a silicon carbide paper (roughness: P4000) to remove overhangs and produce smooth line edges. The total number of specimens was 600 (10 materials × 3 artificial aging protocols × 20 specimens per experimental group). The artificial aging protocols included:

(I) Dark storage in distilled water at 37 °C for 1 day;
(II) Dark storage in distilled water at 37 °C for 30 days;
(III) Dark storage in distilled water at 37 °C for 30 days, followed by thermocycling (10,000 cycles between 5 and 55 °C, dwell time: 30 s).

The specimens were loaded until failure in a universal testing machine (Inspekt Duo 5kN-M; Hegewald & Peschke, Nossen, Germany) using a three-point bending test device with an inter-support span of 12 mm and a crosshead speed of 1 mm/min. Mechanical testing was performed in distilled water at room temperature. FS and FM were calculated according to the following equations:

(1) $$\mathrm{FS}=\frac{3F_{f}l}{2bh^{2}}$$

(2) $$\mathrm{FM}=\frac{F_{l}l^{3}}{4bh^{3}y_{l}}\times \left[1+2.85{\left(\frac{h}{l}\right)}^{2}-0.84{\left(\frac{h}{l}\right)}^{3}\right]$$

where *F_f_* = force at fracture (N), *l* = span between supports (mm), *b* = specimen width (mm), *h* = specimen height (mm), *F_l_* = force at the end of the linear part of the force–deflection diagram (N), and *y_l_* = deflection at the end of the linear part of the force–deflection diagram (mm). In Equation (2), the expression $\left[1+2.85{\left(\frac{h}{l}\right)}^{2}-0.84{\left(\frac{h}{l}\right)}^{3}\right]$ represents the shear correction [33].

### 2.3. Degree of Conversion

**Figure 1:**
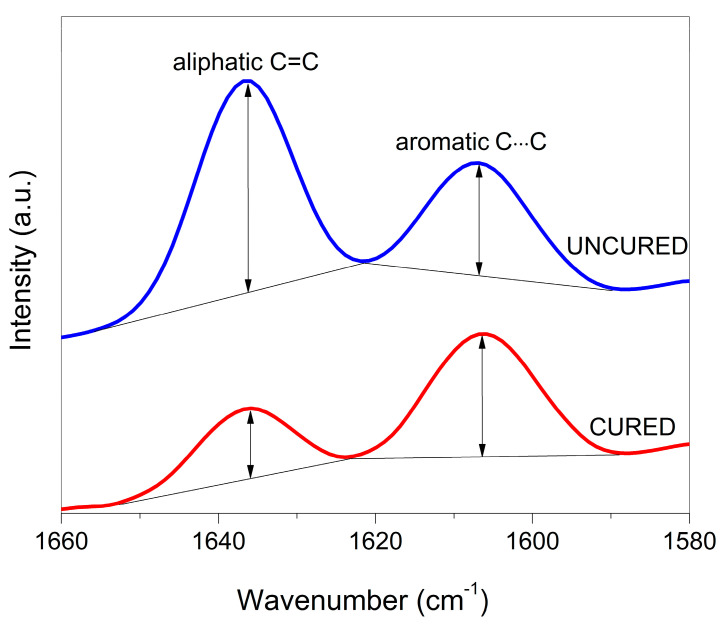
Illustration of Fourier-transform infrared spectra before and after curing that were used for calculating the degree of conversion of experimental composites.

Degree of conversion (DC) measurements were performed on the surface of rectangular specimens prepared in the same manner as previously described for mechanical properties. The specimens were stored dry at 37 °C for 1 day and infrared spectra were collected from the specimen surface that was in contact with PET foil during the light-curing. The middle part of the specimen was pressed against the diamond attenuated total reflectance (ATR) accessory of a Fourier-transform infrared (FTIR) spectrometer (Nicolet iS50, Thermo Fisher, Madison, WI, USA). Due to high radiant exposure of the curing light, illumination from both specimen sides, and light transmittance of the experimental composites typically higher than that of commercial resin composites [34], the surface DC values were considered representative for the whole specimen. FTIR spectra were collected using 50 scans with a resolution of 4 cm^−1^. Figure 1 illustrates relative changes in the FTIR spectrum during composite polymerization. The decrease in intensity of the spectral band at 1638 cm^−1^ that occurs after curing is used to determine DC. The spectral band at 1608 cm^−1^ was used as an internal standard; hence, DC was calculated from the changes in the ratio of absorbance intensities of the spectral bands at 1638 cm^−1^ (aliphatic C=C) and 1608 cm^−1^ (aromatic C⋯C) according to Rueggeberg’s standard baseline method [35], using the following equation:

(3) $$\mathrm{DC}\;\left(\%\right)=\left[1-\frac{{\left(1638\;{\mathrm{cm}}^{-1}/1608\;{\mathrm{cm}}^{-1}\right)}_{peak\;height\;after\;curing}\;}{{\left(1638\;{\mathrm{cm}}^{-1}/1608\;{\mathrm{cm}}^{-1}\right)}_{peak\;heiht\;before\;curing}}\right]\times \;100$$

Six specimens per material were prepared for the DC measurements.

### 2.4. Statistical Analysis

Normality of distribution was verified by Shapiro Wilk’s test and inspection of normal Q-Q plots. For FS and FM, two-way ANOVA was used to evaluate the effects of material composition and artificial aging protocol. Since statistically significant interactions were identified, the statistical analysis was followed by one-way ANOVAs performed for each factor separately. To adjust for multiple comparisons, Tukey post-hoc correction was used. The DC values among the materials were statistically compared using one-way ANOVA with Tukey post-hoc correction. The statistical analysis was performed using SPSS (version 25; IBM, Armonk, NY, USA) with an overall level of significance for all comparisons of 0.05.

The reliability analysis was performed using Weibull statistics by plotting the function ln ln [1/(1 − P_f_)] = m (ln σ − ln σ_θ_), where P_f_ = probability of failure, m = Weibull modulus σ = flexural strength at failure, and σ_θ_ = characteristic strength [36]. In this equation, “m” represents the shape parameter of the Weibull distribution and can be used to evaluate the changes in material reliability after artificial aging. As each experimental group consisted of *n* = 20, Weibull graphs were plotted using 20 data points, which were fitted to a linear function using maximum likelihood estimation [36]. Weibull analysis was performed using OriginPro (version 9.1; OriginLab, Northampton, MS, USA).

## 3. Results

**Figure 2:**
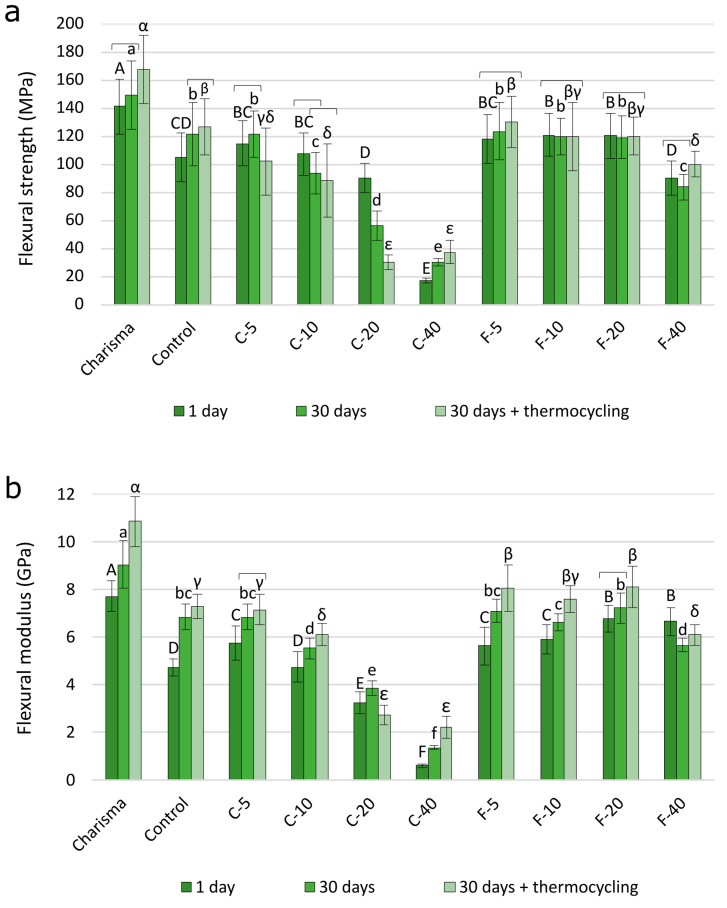
Flexural strength (**a**) and flexural modulus (**b**) presented as mean values ± SD. Statistically similar values among artificial aging protocols are connected with square brackets. Statistically similar values among materials are denoted by same uppercase, lowercase, and Greek letters for the following artificial aging protocols, respectively: 1 day, 30 days, and 30 days + thermocycling.

The results of one-way ANOVA are presented in Table 4. FS and FM values are presented in Figure 2. In the C-series, FS values ranged between 17.1 and 121.5 MPa and were considerably more diminished by the increasing BG amounts than FS values of the F-series (83.8–130.2 MPa). Analogously, FM values in the C-series (0.60–7.16 GPa) were more reduced by the increase in BG amount than in the F-series (5.63–8.13 GPa).

Compared to the Control material, statistically similar or better FS and FM were measured in the F-series for BG filler loading of up to 20 wt%. In the C-series, FS and FM values were significantly lower compared to those of the Control material already for 10 wt% of BG.

The ISO-recommended “minimum acceptable” FS values for restorative resin composites of 80 MPa were achieved for all materials in the F-series, while in the C-series, the materials with higher BG amounts (20 and 40 wt%) failed to meet the requirement of 80 MPa.

The effect of artificial aging on FS was material-dependent, leading to either a statistically significant increase (Charisma, Control, C-40, and F-40), no statistically significant change (F-5, F-10, and F-20), or a statistically significant decrease (C-5, C-10, and C-20). For most materials, FM showed a statistically significant increase with artificial aging, except for two materials that showed a significant decrease (C-20 and F-40).

**Figure 3:**
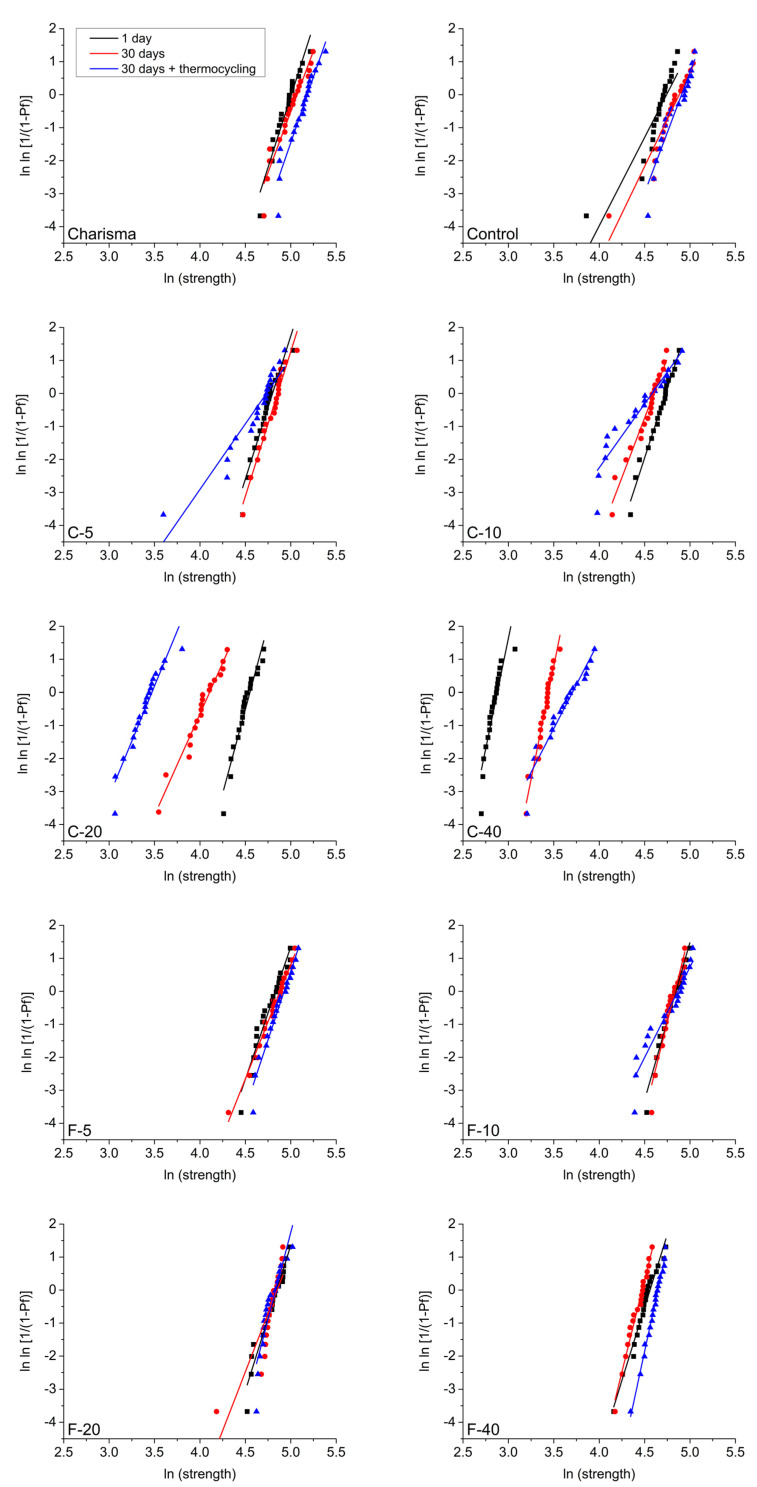
Weibull plots for the analysis of material reliability after artificial aging. Slopes of the fit lines indicate material reliability.

**Figure 4:**
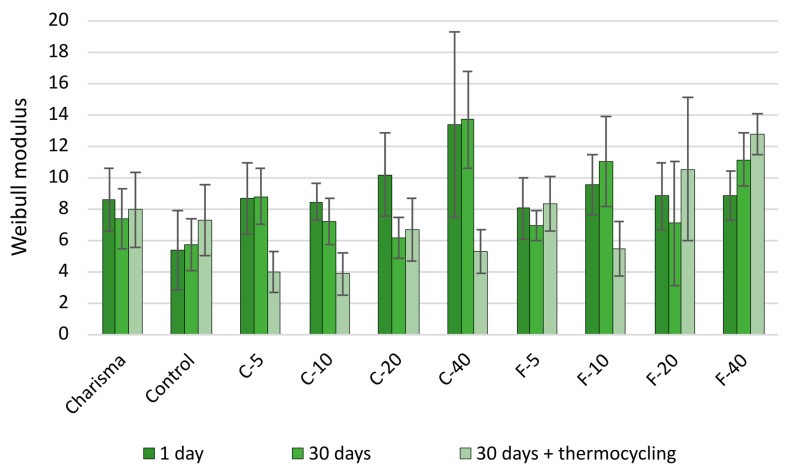
Weibull modulus. Error bars denote 95% confidence interval.

Weibull plots in Figure 3 show the distribution of FS values as a function of probability of failure. A higher slope (higher Weibull modulus) of fit curves indicates a narrower distribution and a more reliable material. The estimated values of the Weibull modulus (Figure 4) can be statistically compared among three artificial aging protocols by observing the overlap between their 95% confidence intervals. The C-series showed a statistically significant decrease in reliability after artificial aging. In contrast, the commercial and experimental references (Charisma and Control) and most of the materials in the F-series (all except F-10) maintained a more consistent reliability.

**Figure 5:**
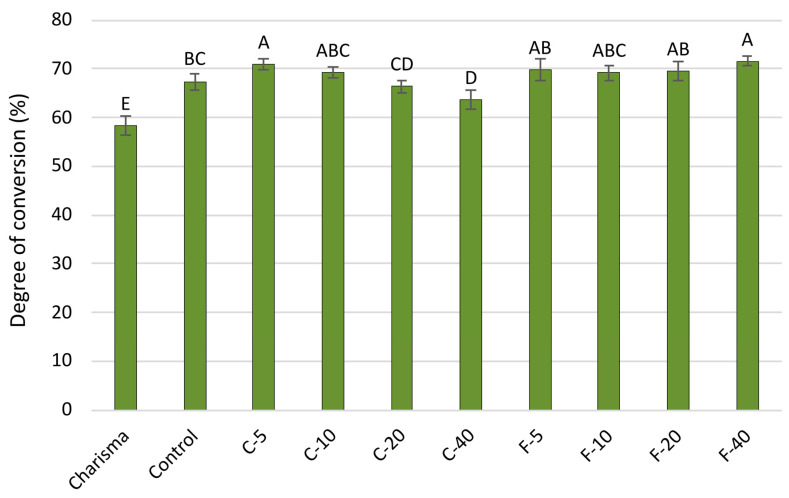
Degree of conversion measured 1 day post-cure (mean values ± SD). Statistically similar values among materials are denoted by same uppercase letters.

The DC values presented in Figure 5 show that all experimental composites had significantly higher DC values than the commercial reference (Charisma). In the F-series, DC values were statistically similar or higher compared to that of the Control composite, while DC in the C-series showed a decline with increasing BG amounts, being significantly lower compared to the Control for the composite containing 40 wt% of BG.

## 4. Discussion

This study investigated the FS and FM of experimental composites filled with a customized low-Na F-containing BG that was envisioned as a more stable alternative to the conventional BG 45S5. All three research hypotheses were accepted since the flexural properties were significantly affected by BG type, BG amount, and artificial aging. The composites functionalized with 5–40 wt% of the customized low-Na F-containing BG (F-series) showed significantly improved mechanical properties compared to the composites with corresponding amounts of the conventional BG 45S5 (C-series). The accompanying FTIR analysis indicated that the reduction of mechanical properties in the C-series could be partly attributed to the DC reduction resulting from the addition of BG 45S5.

Under clinical conditions, the exposure of restorative resin composites to an aqueous environment begins shortly after light-curing and lasts throughout the service life of the restoration. In conventional resin composites, which contain only reinforcing silanized fillers, hydrolytic degradation dominantly occurs within the polymer network or the filler/polymer interface, whereas the inorganic part of the resin composite (silanized glass filler) is less susceptible to hydrolytic breakdown [37]. In contrast, ion-releasing materials contain unsilanized reactive fillers, which are water-soluble by design and hence represent the weakest link of the material structure [38]. Soluble BG fillers diminish the initial mechanical properties of the composite by being incapable of chemical bonding with the polymer matrix, while during material aging in an aqueous environment, these fillers contribute to further degradation of mechanical properties by making the composite more hydrophilic and allowing higher water uptake [39], which in turn leads to a more pronounced degradation of both the polymer matrix and the interface between the polymer matrix and reinforcing fillers. In this way, the components of the material, which are in conventional inert composites otherwise protected from excessive breakdown by the material’s hydrophobic nature, are made more sensitive to degradation.

Despite the fact that laboratory simulations of material aging cannot simulate all complexities of the mechanical, chemical, thermal, and microbiological factors affecting material degradation under clinical conditions, artificial aging is useful for evaluating material properties after its structure has been permeated with water (or some other solution) and consequently underwent the effects of hydrolysis, plasticization, and repeated stressing of the filler/matrix interface [22]. Today’s resin composites are known to perform favorably shortly after curing, whereas a more realistic mechanical behavior is seen only after artificial aging [40]. Hence, regardless of the selection of artificial aging protocol, simulated degradation of the material helps to obtain values of mechanical properties that are more representative of a realistic composite restoration. This aspect is especially important for ion-releasing composites that undergo a more extensive degradation compared to their inert counterparts [7].

Previous studies have reported inconsistent and material-dependent effects of artificial aging on the mechanical properties of experimental composites functionalized with BG. Al-eesa et al. showed a significant FS reduction after 84 days of immersion in artificial saliva for experimental composites filled with 80 wt%/58 vol% of low-Na BG (either silanized or unsilanized) [7]. These composites contained no other reinforcing inert fillers; additionally, the BG filler size was reported as “below 38 microns”, which is larger than the usual filler size in contemporary composites (around 1 micron). A similar pattern of a significant FS/FM decrease with artificial aging in water and ethanol was reported in a study on experimental composites filled with 5–40 wt% of smaller particles (d50/d99: 4.0/13.0 μm) BG 45S5 combined with reinforcing inert fillers up to 70 wt% of total filler load [13]. In contrast, no significant change in FS after 2 months of immersion in *S. mutans* culture was found for composites filled with 5–15 wt% of Na-free BG [41]; no significant changes in FS and FM were also reported after 3-month storage at neutral or acidic pH for the commercial remineralizing material Cention (containing 30–40 wt% of low-Na, phosphate-free BG) [42]. In a study on experimental composites functionalized with copper-doped mesoporous BG nanospheres, aging-induced degradation of FS and FM depended on the variations in the amounts of functional and reinforcing fillers [43]. The different outcomes observed in the aforementioned studies are due to considerable differences in the composition, particle size, and amount of BG fillers, as well as different resin matrices and artificial aging methods. Although not much generalization can be made based on such highly heterogeneous studies, their results suggest that it is indeed possible to formulate BG-functionalized composites with improved resistance to aging-induced degradation. The F-series containing the customized low-Na F-containing BG in the present study appears to belong to such a group of materials.

The ISO guidelines for restorative resin composites feature simplified testing procedures, which are intended for fast and cost-effective screening of fundamental properties. Hence, the ISO-recommended protocol for FS/FM testing omits artificial aging and requires that material tested after 1 day of water storage should show flexural strength of at least 80 MPa to be regarded clinically acceptable. The 1-day period is supposed to allow the composite to finish the post-cure polymerization, partially absorb water, and relieve residual internal stresses. In this way, the ISO recommendation assumes that the composite that demonstrates 80 MPa 1 day after curing would maintain acceptable mechanical properties over its service life, ideally lasting for several decades. While this reasoning may be valid for commercial composites, the ion-releasing composites are likely to undergo much more extensive degradation with aging. For example, C-20 showed a three-fold FS decrease after artificial aging (from 90 MPa to 30 MPa), indicating that the ISO testing protocol and the stipulated criteria are less well adapted to the composites that contain reactive fillers. Nonetheless, all of the materials in the F-series surpassed the 80 MPa threshold even after thermocycling, indicating considerably better stability compared to the materials functionalized with the same amounts of BG 45S5. Even the worst-performing material from the F-series (F-40) with FS in the range of 84–100 MPa showed no statistically significant degradation of mechanical properties with aging. The other materials from the F-series (with 5–20 wt% of BG) demonstrated rather constant FS values of about 120 MPa, regardless of artificial aging.

The statistically significant increase in FM due to artificial aging observed in most of the tested materials (all materials except C-20 and F-40) may seem surprising since basic mechanical properties such as FS and FM are generally expected to degrade with storage in water. However, in resin composite specimens that are immersed in water immediately after curing, material “maturation” due to post-cure polymerization occurs simultaneously with water sorption. Hence, the resulting flexural properties are dependent on the relationship between the continued strengthening of the matrix through the post-cure polymerization and the water-induced degradation. If degradation is not too pronounced, it is possible that the post-cure reaction results in a net improvement of mechanical properties over the first month of aging [44]. This effect has been reported for commercial composites, which showed a significant increase in FM after 30 days of immersion in water at 37 °C [45]. Indeed, resin systems based on bis-GMA, such as that used in our study, have been reported to be resistant to water-induced degradation [46], giving credibility to the idea that the balance between degradation and post-cure polymerization was shifted in favor of the latter. The observed FM increase with aging is additionally supported by the fact that elastic modulus is not particularly affected by the lack of surface silanization of BG fillers, since it is determined by the direct physical contact of particles with the resin matrix, being less dependent on the quality of the interface [11]. On the other hand, FS is more dependent on the strength of the filler/matrix interface, which explains why an effect similar to the post-cure improvement in FM was not also observed for FS of the BG-containing composites. Lastly, it has also been proposed that water immersion can improve the elastic modulus of resin composite materials by facilitating the removal of residual monomers that act as plasticizers, thus allowing the polymerization to continue and enhance crosslinking [47]. This mechanism presumably becomes more important as the DC of the material decreases (i.e., more residual monomers) and may explain why for the material with the lowest DC (C-40) artificial aging significantly increased not only FM but also FS.

The reduction of DC as a result of adding BG 45S5 to the experimental composites has been reported in several previous studies in which it was attributed to the inhibition of free-radical polymerization by oxides on particle surfaces or the inhibitory effect of water present on the surface of hygroscopic BG 45S5 [48,49,50]. The DC reduction due to the increasing amounts of BG 45S5 in the C-series reflected on mechanical properties measured initially (after 1 day), as well as after artificial aging. The short-term (1 day) reduction in FS and FM occurred because the polymeric network with lower DC is mechanically weaker and more deformable, whereas after artificial aging, FS and FM were additionally impaired by enhanced water sorption into the less polymerized resin. Besides negatively affecting the DC reached during light-curing, BG 45S5 has been reported to diminish the post-cure polymerization [48], hence limiting the improvement of mechanical properties with aging. This may be the reason why in the C-series, the post-cure polymerization was unable to compensate for the reduction of mechanical properties due to water sorption. A different development of mechanical properties observed in the F-series, which maintained FS and FM significantly better over artificial aging, is due to the lack of the inhibitory effect on polymerization of photocurable bis-GMA/TEGDMA resins shown by the customized low-Na F-containing BG, as demonstrated in previous studies [25,34,51]. Consequently, the F-series not only had a more complete initial polymerization but was also able to continue the post-cure polymerization during artificial aging.

Another process that has been hypothesized to affect the flexural properties of resin composites is the development of hoop stresses around filler particles due to polymerization shrinkage [52]. On the one hand, these internal residual stresses may diminish flexural properties, while on the other hand, their direction toward particle surfaces can improve the strength of the filler/matrix interface by making filler particles more difficult to pull out from the polymer. The hoop stresses likely develop differently around silanized reinforcing fillers that are chemically bonded to the matrix and unbonded BG fillers that remain free within the voids in the matrix. As the relative ratio of unbonded to bonded filler particles increases with the increasing BG amounts, the buildup of internal stresses can be expected to change, although it is unclear in which direction. Hence, the observed variations in the mechanical properties among the experimental composites were likely affected by the complex interplay of the factors discussed above, namely, (I) the continuing post-cure polymerization, (II) the fact that the polymerization was inhibited in the C-series as a function of increasing amounts of BG 45S5, (III) the two-fold effect of internal hoop stresses on mechanical properties, (IV) the supposed dependence of residual stresses on the ratio of bonded/unbonded fillers, and (V) the partial relaxation of residual stresses with water sorption.

In addition to mean values of mechanical properties evaluated in the conventional ANOVA, material reliability is another important parameter that is useful for describing the predictability of material when exposed to mechanical loading, i.e., low reliability indicates that the material tends to fail at a wide range of stress values, which is an unfavorable behavior despite the mean value of stress at failure may be high. Vice versa, high reliability indicates that the material is more predictable because it fails within a narrower range of stress values. By performing a sufficient number of repeated experimental runs, the probability of failure can be evaluated as a function of stress at failure using Weibull analysis, which makes use of the shape parameter of the Weibull distribution to describe how predictably the material fails under mechanical loading [53]. The material reliability evaluated by Weibull analysis depends on the distribution of crack-initiating flaws within the tested specimen. These flaws may be air bubbles, imperfections on the filler/resin interface, or in the case of unsilanized BG particles, the complete lack of bonding at the filler/resin interface. The filler/resin interface at the surface of BG particles is capable of stress transfer from the matrix to the filler particles only through direct physical contact with the resin matrix. This direct contact is lost when due to immersion in water the outer layer of filler particles is dissolved; this causes the spaces occupied by BG particles to behave as structural flaws. The extent of degradation by this mechanism is determined by the reactivity of a particular BG composition; hence, the more reactive BG 45S5 in the C-series produced a significant decrease in material reliability with aging, whereas the F-series functionalized with the less reactive customized low-Na BG maintained more stable reliability values.

When considering the effect of BG amount on material reliability, the values of Weibull modulus should be interpreted in context of mean FS values. For example, the results obtained after 1 day show that the increase in the BG amount from 5 to 40 wt% in the C-series led to a steady trend of increase in Weibull modulus but also to a 6.7-fold decrease in FS. In this case, the narrowing of the interval at which specimens fractured does not contribute to clinical usability of the material.

## 5. Conclusions

This in vitro study that compared the effects of functionalizing resin composites with 5–40 wt% of conventional bioactive glass 45S5 and a customized low-Na F-containing bioactive glass on flexural strength and modulus concluded the following:

1. Mechanical properties were significantly less impaired by the customized bioactive glass;
2. The reduction of mechanical properties with aging was diminished for the customized bioactive glass;
3. Flexural strength values recommended by the ISO 4049 standard (80 MPa) were attained for all filler loadings (5–40 wt%) of the customized bioactive glass, whereas for the conventional bioactive glass 45S5 the same criterion was fulfilled only for filler loading of up to 20 wt%.

## Figures and Tables

**Table 1:** Composition of inorganic fillers used in experimental resin composites.

	Bioactive Glass 45S5	Low-Sodium Fluoride-Containing Bioactive Glass	Inert Barium Glass	Silica
Particle size (d50)	3 µm	3 µm	1 µm	5–50 nm
Composition (wt%)	45.0% SiO_2_ 24.5% CaO 24.5% Na_2_O 6.0% P_2_O_5_	33.5% SiO_2_ 33.0% CaO 10.5% Na_2_O 11.0% P_2_O_5_ 12.0% CaF_2_	55.0% SiO_2_ 25.0% BaO 10.0% Al_2_O_3_ 10.0% B_2_O_3_	>99.8% SiO_2_
Silanization (wt%)	none	none	3.2	4–6
Manufacturer	Schott, Mainz, Germany	Schott, Mainz, Germany	Schott, Mainz, Germany	Evonik, Hanau, Germany
Product name/LOT	G018-144/M111473	experimental batch	GM27884/Sil13696	Aerosil R 7200/157020635

**Table 2:** Properties of methacrylate resins used in experimental resin composites.

	Bisphenol A-Glycidyl Methacrylate (Bis-GMA)	Triethylene Glycol Dimethacrylate (TEGDMA)
CAS Number	1565-94-2	109-16-0
Molecular formula	C_29_H_36_O_8_	C_14_H_22_O_6_
Molar mass (g/mol)	512.60	286.32
Refractive index (at 25 °C)	1.540	1.4595
Viscosity (Pa·s)	910	0.01

**Table 3:** Composition of experimental resin composites.

Material Designation		Filler Composition (wt%)			Total Filler Ratio (wt%)
		Bioactive Glass 45S5	Experimental Low-Sodium Fluoride-Containing Bioactive Glass	Reinforcing Fillers (Inert Barium Glass/Silica = 2:1)	
	Control	0	0	70	70
C-series	C-5	5	0	65	70
	C-10	10	0	60	70
	C-20	20	0	50	70
	C-40	40	0	30	70
F-series	F-5	0	5	65	70
	F-10	0	10	60	70
	F-20	0	20	50	70
	F-40	0	40	30	70

**Table 4:** Results of one-way ANOVA representing *p*-values and partial eta-squared values for the comparisons of artificial aging protocols.

	Flexural Strength		Flexural Modulus	
Material	*p*-Value	Partial Eta Squared	*p*-Value	Partial Eta Squared
**Charisma**	0.0019	0.1978	0.0000	0.6750
**Control**	0.0034	0.1811	0.0000	0.8458
**C-5**	0.0078	0.1565	0.0000	0.4852
**C-10**	0.0060	0.1642	0.0021	0.1945
**C-20**	0.0000	0.8260	0.0001	0.2704
**C-40**	0.0000	0.7417	0.0000	0.8610
**F-5**	0.1429	0.0660	0.0000	0.6356
**F-10**	0.9828	0.0006	0.0000	0.6410
**F-20**	0.9618	0.0014	0.0000	0.3914
**F-40**	0.0000	0.3094	0.0000	0.4556

## Data Availability

Datasets are available from the corresponding author on reasonable request.
